# Lifestyle-integrated functional exercise to prevent falls and promote physical activity: Results from the LiFE-is-LiFE randomized non-inferiority trial

**DOI:** 10.1186/s12966-021-01190-z

**Published:** 2021-09-03

**Authors:** Carl-Philipp Jansen, Corinna Nerz, Sarah Labudek, Sophie Gottschalk, Franziska Kramer-Gmeiner, Jochen Klenk, Judith Dams, Hans-Helmut König, Lindy Clemson, Clemens Becker, Michael Schwenk

**Affiliations:** 1 Network Aging Research, Heidelberg University, Bergheimer Strasse 20, 69115 Heidelberg, Germany; 2 Department of Clinical Gerontology and Geriatric Rehabilitation, Robert Bosch Hospital, Stuttgart, Germany; 3 Department of Health Economics and Health Services Research, University Medical Center Hamburg-Eppendorf, Hamburg, Germany; 4 Institute of Epidemiology and Medical Biometry, Ulm University, Ulm, Germany; 5 IB University of Health and Social Sciences, Study Centre Stuttgart, Stuttgart, Germany; 6 Faculty of Health Sciences, University of Sydney, Sydney, Australia

**Keywords:** Fall prevention, Non-inferiority trial, Fall risk, Intervention costs, Physical activity promotion

## Abstract

**Background:**

The ‘Lifestyle-integrated Functional Exercise’ (LiFE) program successfully reduced risk of falling via improvements in balance and strength, additionally increasing physical activity (PA) in older adults. Generally being delivered in an individual one-to-one format, downsides of LiFE are considerable human resources and costs which hamper large scale implementability. To address this, a group format (gLiFE) was developed and analyzed for its non-inferiority compared to LiFE in reducing activity-adjusted fall incidence and intervention costs. In addition, PA and further secondary outcomes were evaluated.

**Methods:**

Older adults (70 + years) at risk of falling were included in this multi-center, single-blinded, randomized non-inferiority trial. Balance and strength activities and means to enhance PA were delivered in seven intervention sessions, either in a group (gLiFE) or individually at the participant’s home (LiFE), followed by two “booster” phone calls. Negative binomial regression was used to analyze non-inferiority of gLiFE compared to LiFE at 6-month follow-up; interventions costs were compared descriptively; secondary outcomes were analyzed using generalized linear models. Analyses were carried out per protocol and intention-to-treat.

**Results:**

Three hundred nine persons were randomized into gLiFE (*n* = 153) and LiFE (*n* = 156). Non-inferiority of the incidence rate ratio of gLiFE was inconclusive after 6 months according to per protocol (mean = 1.27; 95% CI: 0.80; 2.03) and intention-to-treat analysis (mean = 1.18; 95% CI: 0.75; 1.84). Intervention costs were lower for gLiFE compared to LiFE (-€121 under study conditions; -€212€ under “real world” assumption). Falls were reduced between baseline and follow-up in both groups (gLiFE: -37%; LiFE: -55%); increases in PA were significantly higher in gLiFE (+ 880 steps; 95% CI 252; 1,509). Differences in other secondary outcomes were insignificant.

**Conclusions:**

Although non-inferiority of gLiFE was inconclusive, gLiFE constitutes a less costly alternative to LiFE and it comes with a significantly larger enhancement of daily PA. The fact that no significant differences were found in any secondary outcome underlines that gLiFE addresses functional outcomes to a comparable degree as LiFE. Advantages of both formats should be evaluated in the light of individual needs and preferences before recommending either format.

**Trial registration:**

The study was preregistered under clinicaltrials.gov (identifier: NCT03462654) on March 12^th^ 2018

**Supplementary Information:**

The online version contains supplementary material available at 10.1186/s12966-021-01190-z.

## Background

Tackling physical inactivity and mobility disability in the face of wide-spread sedentariness has been declared a paramount objective which is founded on the numerous positive effects on health and aging-associated morbidity in older persons [1, 2]. Physical activity (PA) further improves independence, increases participation, and enhances quality of life [3, 4]. However, PA comes with certain risks. Every transition and step increase the risk of falling, which holds true particularly when performed unsafely by older adults with low functional capacity [5–8]. Recommendations to increase PA in older persons have often neglected this potential trade-off. Especially walking has been recognized as a “hazardous” PA in older persons [9, 10]. Arguably, beneficial effects of high PA may outweigh this risk by maintenance or improvement of functional capacity and mobility in the midterm, but oversimplified recommendations for increasing PA may not be unconditionally appropriate for the older population. Novel interventions should therefore be tested looking at an increase of safe PA, being defined as, e.g., falls per one million steps or falls per distance walked [11]. This requires sensor-based measurements of PA and mobility alongside classical outcome assessments of functional performance and perceived function. In summary, interventions should increase PA and simultaneously prevent falls and fall-related injuries. Examinations of a combined endpoint of fall risk and activity have been proposed as the gold standard approach [11–13].

The ‘Lifestyle-integrated Functional Exercise’ (LiFE) trial is a landmark study in this respect, having looked at both PA and falls [14], although without combining these endpoints for analysis. The study had shown significant improvement of balance and strength capacity of older persons aged 70 + years and further promoted an increase of PA [15]. A possible downside of LiFE in certain settings is that it is delivered individually by therapists in seven home visits. This comes with considerable human resources and costs. A smaller pilot trial from Canada raised the idea that a group-based LiFE format may be similarly effective [16]. This was supported by findings from a feasibility study evaluating the group-based LiFE (gLiFE) program used in this trial [17]. The economical assumption, that a group format could be less costly, as well as the group format’s effectiveness in terms of the abovementioned combined endpoint have yet to be investigated.

Therefore, a non-inferiority trial was carried out to evaluate whether a group-based LiFE (gLiFE) program is not less effective than the original LiFE program (LiFE) by more than an acceptable amount while being less costly in terms of intervention costs. The acceptable amount is a predefined non-inferiority margin for the treatment effect in the trial’s primary outcome [18]. Non-inferiority investigations require that the reference treatment’s efficacy is established [18]. Given the high quality of the LiFE trial and the positive effects found [14], we considered this prerequisite confirmed.

Primary objectives of this study were 1) to compare non-inferiority of gLiFE compared to LiFE in reducing activity-adjusted fall incidence; and 2) to compare intervention costs of both formats. The corresponding hypotheses were that gLiFE is not less efficacious than LiFE in reducing activity-adjusted fall incidence, and that its delivery is less costly compared to LiFE. The secondary objective was to compare effectiveness of both formats regarding functional (dis-)ability, adherence, motor capacity, fall-related outcomes, fear of falling, balance confidence, and adverse events.

## Methods

### Study design

This study (“LiFE-is-LiFE”) was a multi-center, single-blinded, randomized non-inferiority trial conducted in Heidelberg and Stuttgart, Germany. The full study protocol is available elsewhere [19]. The study was preregistered under clinicaltrials.gov (identifier: NCT03462654) on March 12^th^ 2018. Reporting in this article is aligned with the CONSORT extension in non-inferiority trials [18] [see CONSORT checklist, Additional file 3].

In addition to baseline assessment, follow-up assessments were carried out six and twelve months after intervention start (reference was the date of the first (g)LiFE session), with a tolerance of ± 2 weeks.

### Participants and eligibility criteria

**Fig. 1:**
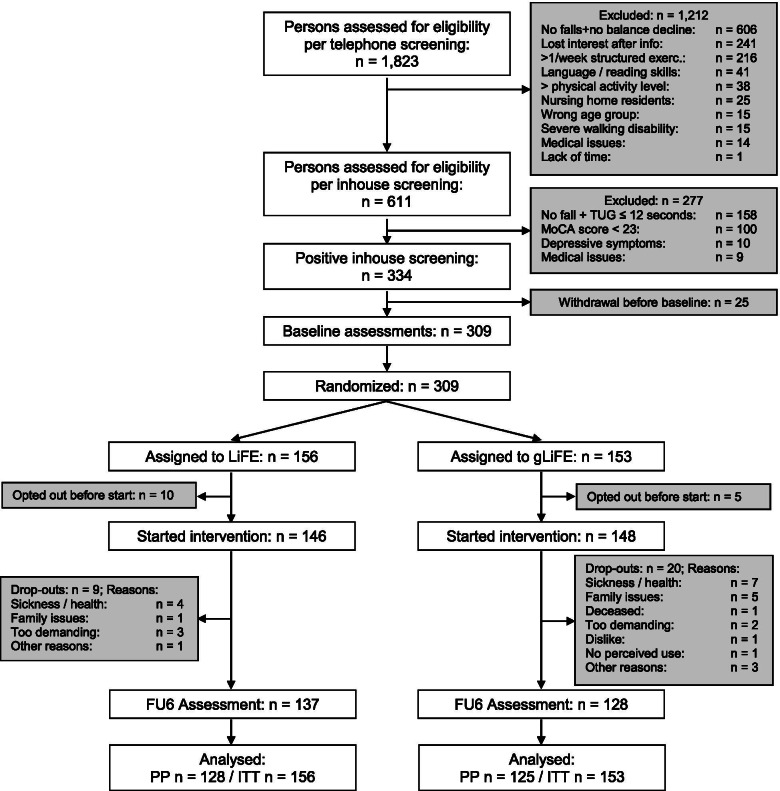
Participant flow; FU6: 6-month follow-up assessment; PP: per protocol; ITT: intention-to-treat; MoCA: Montreal Cognitive Assessment; TUG: Timed Up-and-Go

For recruitment purposes a list of all persons aged 70 +  was drawn from municipality registries in both cities. Persons were drawn consecutively in waves of between 250 and 1.000 persons and contacted between April 2018 and July 2019 by mail. If interested, participants could contact the study sites for a first eligibility screening by telephone. In case of a positive telephone screening, an inhouse screening was scheduled. Participant flow is depicted in Fig. 1. To be included in the study, participants had either a) experienced at least one injurious or more than one non-injurious fall in the year prior to study participation according to self-report, or b) were designated as having high risk of falls when indicating self-perceived balance decline and needing ≥ 12 s for the “Timed Up-and-Go” (TUG) [20] test. Those who already exercised more than once per week or indicated to carry out more than 150 min of moderate to vigorous PA per week were excluded. A detailed list of further exclusion criteria is provided in the study protocol [19].

### Randomization and blinding

Participants were randomized after baseline assessment into one of the two intervention arms through block-randomization. Apart from block sizes, randomization was concealed, i.e., staff was not aware of the sequence before randomization. Randomization and group assignment was carried out by the study site coordinators (CPJ, CN) in an externally managed database without possibility to alter group allocation afterwards. In case of withdrawal from intervention, participants were still eligible for follow-up assessments. Assessors were blinded to group allocation at all times.

### Intervention programs

A detailed description including a TIDieR checklist of both intervention formats is included in the study protocol [19]. In the LiFE program, balance and strength activities as well as general PA promoting activities are embedded into everyday tasks and routines, with the overall aim to integrate them in a way that these activities can be performed multiple times a day [14]. As there was no standardized group format of the LiFE program available, gLiFE had been developed according to Medical Research Council guidelines [21] and piloted in advance to the intervention start [17]. In both intervention arms, intervention components were taught in accordance with the LiFE trainer’s manual [15], including strength and balance activities as well as strategies to enhance physically active behavior and to habitualize activities as part of individual daily routine. LiFE and gLiFE were delivered in seven sessions within eleven weeks, either in a group (gLiFE) or at the participant’s home (LiFE), followed by two booster phone calls in week four and ten after the last intervention session. During the intervention sessions a total of seven balance activities, seven strength activities for the lower extremities, and two PA promoting activities were delivered. To help participants establish a LiFE routine as part of their daily life, they learned how to independently select, execute, and adapt intensity of activities, and how to identify appropriate daily situations in which LiFE activities can be integrated. gLiFE group sessions were scheduled for two hours and held by two trainers with a maximum of 12 participants; LiFE sessions lasted approximately one hour and were delivered by one trainer. Trainers were either physio therapists, sports scientists, health psychologists, or occupational therapists who had attended a two-day workshop prior to the start of the intervention delivery, including a certification test.

### Outcomes

#### Primary outcomes

*Combined endpoint: Falls adjusted for PA.* To measure PA, “activPAL4™ micro” accelerometers (PAL Technologies Ltd., Glasgow, Scotland) were attached to participants’ central front thigh at baseline, 6-, and 12-month follow-up to continuously measure PA under “free-living” conditions for seven days (24 h), i.e., activPALs were posted back to the respective study centers no earlier than the start of the ninth day of measurement. The sensor was wrapped in a nitrile finger cot fixed with a waterproof, adhesive, transparent film. The device has shown good to excellent reliability and validity [22]. If the device was removed earlier, data were used if at least two weekdays and the Sunday of the respective week were fully captured [23]. Given that walking activity can be seen as the most hazardous PA when it comes to risk of falling [9, 10], PA exposure was operationalized as mean steps/day.

Falls were defined as “an unexpected event in which the participant comes to rest on the ground, floor, or lower level” [24] and were recorded using a monthly falls calendar sent back by use of preaddressed and prestamped envelopes. In case of a fall, information on location, date, time, injuries, subsequent treatment related to the fall, and movement during which the person has fallen had to be provided on the calendar sheet. Following recommendations of Gillespie et al. [25], falls were followed-up via telephone calls to ascertain additional information and to determine the current health status of the person.

*Intervention costs*. Intervention costs were calculated as costs per participant for each group (LiFE/gLiFE). Personnel and material costs, trainers’ and participants’ travel expenses, and room rent were taken into account. The average group size of gLiFE sessions was 7.9 persons. The duration of the sessions (including time for travel and preparation) resulted in 1.8 (LiFE) and 3.0 (gLiFE) personnel hours per session. Personnel costs per hour were derived from the German wages agreement for civil services 2018 (“TVöD” salary level E13 and E10). Costs for materials, manuals, and working books were considered. Moreover, a room rent of €50 per day for the trainer workshop or per gLiFE session in one of the study centres was also taken into account. In the other study centre, a suitable room was available on site for the gLiFE sessions, therefore no room rent was incurred there.

Since study conditions deviate from conditions in case of an implementation in the “real world”, interventions costs were also calculated for another scenario, based on assumptions that the project team considers to most realistically represent the implementation conditions. In this “real world” scenario, it was assumed that 20 trainers with a salary according to “TVöD” salary level E8 participate in the trainer workshop. It was assumed that on average 12 persons attend the gLiFE sessions and that each gLiFE trainer pair would conduct 12 training sessions per week, while one LiFE trainer could conduct 15 sessions in the same time. The duration of the LiFE/gLiFE sessions (including time for travel and preparation) and phone calls were assumed to be 2.0 h/2.5 h and 0.5 h, respectively. For both interventions, no room rent was assumed. Furthermore, each trainer or trainer pair was assumed to have their own material set. The data and assumptions underlying the calculations of each scenario are summarized in an additional table [see Additional file 1].

#### Secondary outcomes

*Physical activity*. Mean steps/day were assessed to serve as offset variable in the primary outcome analysis to adjust falls for PA, and as PA outcome in itself.

*Fall outcomes*. Falls were assessed and defined according to Lamb et al. [24], that is, number of falls, fall rate per (half) person year, time to event (either fall or end of observation), number of fallers, and frequent fallers (i.e., more than one fall in the past six months). Fall consequences were categorized into minor, moderate or serious injuries according to a standardized system incorporating symptoms as well as medical care use [26].

*Motor capacity. *Gait performance was measured in terms of 7 m gait speed at comfortable and fast pace. The 30 s chair rise was used to evaluate functional leg strength [27]. Static balance was assessed using the adjusted eight level balance scale developed by Clemson et al. [14].

*Functional (dis-)ability*. The Late Life Function and Disability Instrument (LLFDI) was used to assess participants’ difficulties in performing 32 different upper and lower extremity physical activities and actions as well performance of another 16 socially defined life tasks.

*Adherence*. We followed the consensus agreement by Hawley-Hague et al. [28] who recommend reporting adherence in terms of completion (attendance of at least > 75% of sessions is defined as completion [28]), attendance (percentage of sessions attended out of the actual number of sessions), and duration adherence (adherence to predefined LiFE activities at home, assessed using the Exercise Adherence Rating Scale (EARS) [29]). The EARS ranges from 0 to 24.

*Fear of falling and balance confidence*. Participants’ fear of falling was assessed using the Short Falls Efficacy Scale-International [30], a self-rating scale including 7 items ranging from ‘not at all concerned’ (1 point) to ‘very concerned’ (4 points) and resulting in values between 7 (‘not concerned about falling’) and 28 points (‘very concerned about falling’). The Activities-specific Balance Confidence Scale (ABC) was used to measure participants’ confidence in maintaining their balance while performing certain daily activities.

For participants’ characteristics, age, sex, body-mass index, number of medications, number of comorbidities, falls in the past six months, and cognitive status (Montreal Cognitive Assessment) were assessed.

### Sample size and non-inferiority margin

Sample size was calculated based on 12 month data from the original LiFE study [14]; information on this calculation can be found in the study protocol [19]. As outlined in the limitations section COVID-19-induced changes have been made to the methods used for the present analyses. We used 6-month instead of 12-month data to determine non-inferiority of the primary outcome falls per PA. However, we kept the non-inferiority margin (∆) as stated in the study protocol [19], that is, we accept a 20% difference in this outcome as a comparable reduction. As intervention costs of gLiFE are expected to be lower than of LiFE, no non-inferiority margin is defined for this outcome.

### Statistical analyses

The analyses were carried out according to both the intention-to-treat principle (ITT) and the per-protocol principle (PP) to determine the robustness of the results due to missing values [31]. As dates were fixed for the gLiFE sessions, it was expected that some participants might be unable to attend all seven sessions. Therefore, attendance of a minimum of five sessions per participant was preset to assign participants to the PP sample. In accordance with the ITT principle, all randomized participants who completed baseline assessment were included, regardless of whether they had completed the intervention or prematurely dropped out of the study. In addition to missing information due to drop-out, there was occasional missing information in cases that otherwise completed the follow-up assessment. Overall, the percentage of missing values varied between 0 and 17% across different variables. As imputation of missing values is recommended for missing rates above 5% [32], missing data were imputed using multiple imputation by chained equations (MICE) with predictive mean matching as imputation method [33]. In total, 10 datasets were created based on data from baseline and 6-months’ follow-up assessments and analysed separately. Rubin’s rules [34] were applied to pool results from each dataset.

Negative binomial regression was used to compare incidence rate ratios (IRR) of falls between gLiFE and LiFE,taking into account possible overdispersion. In the model for the combined endpoint–falls per PA–mean steps/day were log-transformed and incorporated as exposure variable (offset). Confidence intervals for explorative comparison of changes between baseline and 6-month follow-up in secondary outcomes were obtained using a generalized linear model with repeated measures.

For the primary outcome, non-inferiority was indicated if the upper limit of the two-sided 95% confidence interval (CI) for gLiFE remained below the relative margin (∆) of 20% from LiFE (IRR = 1.20).

Analyses were performed using SPSS (IBM Corp. Released 2020. IBM SPSS Statistics for Windows, Version 27.0. Armonk, NY: IBM Corp). Multiple imputation of missing values was performed using STATA/SE 16.0 (StataCorp. 2019. Stata Statistical Software: Release 16. College Station, TX: StataCorp LLC).

## Results

### Participant flow and baseline characteristics

We randomized 309 persons from June 2018 to July 2019 into gLiFE (*n* = 153) and LiFE (*n* = 156), of which 15 persons dropped out before the start of the intervention. At six months, 44 observations (14.2%) were lost to follow-up, *n* = 25 in gLiFE (16.3%) and *n* = 19 in LiFE (12.2%), respectively. Of the envisaged sensor-based 7-day PA measurement, at least 6 full days were completed by 99.0% of the participants at baseline and 98.8% at follow-up. Similarity of baseline values indicates successful randomization (Table 1). The majority of participants was female; on average, participants were cognitively intact, were moderately active, had mediocre motor function, and rather low fear of falling. No study-associated serious adverse events were reported. Of the 3 study-associated adverse events, all had mild consequences: one fall occurred on the way to an assessment (LiFE), one on the way to a gLiFE session, and one during a participant’s demonstration of his LiFE execution at home while a trainer was present (LiFE).

**Table 1:** Participant characteristics at baseline according to ITT analyses

N (mean ± SD)	All *N* = 309	LiFE *N* = 156	gLiFE *N* = 153
Age, years	78.8 ± 5.3	78.8 ± 5.2	78.7 ± 5.4
Sex, n (%) female	227 (73.5)	115 (73.7)	112 (73.2)
BMI [kg/m^2^]	27.2 ± 4.9	27.7 ± 5.0	26.8 ± 4.7
No. of medications	4.9 ± 3.4	5.0 ± 3.3	4.8 ± 3.4
No. of comorbidities	2.5 ± 1.6	2.5 ± 1.5	2.5 ± 1.6
MoCA Score	26.0 ± 2.0	26.1 ± 2.0	25.9 ± 2.0
No. of steps/day	5,659 ± 2,919	5,778 ± 3,009	5,538 ± 2,828
No. of falls p.p. in past 6 months	0.66 ± 1.1	0.66 ± 1.1	0.65 ± 1.1
% of fallers in past six months	126 (40.8)	63 (40.4)	63 (41.2)
LLFDI Function	57.3 ± 7.9	57.4 ± 8.0	57.3 ± 7.9
LLFDI Frequency	49.4 ± 4.3	49.3 ± 4.2	49.5 ± 4.4
LLFDI Disability	70.7 ± 12.0	71.7 ± 12.3	69.6 ± 11.5
Gait speed comfortable [m/s]	1.03 ± 0.20	1.03 ± 0.20	1.03 ± 0.21
Gait speed fast [m/s]	1.40 ± 0.32	1.37 ± 0.29	1.43 ± 0.35
30 s Chair Stand	9.1 ± 3.9	9.2 ± 3.8	9.0 ± 3.3
8 Level Balance Scale	4.3 ± 1.5	4.2 ± 1.5	4.4 ± 1.4
Short FES-I	10.4 ± 3.0	10.4 ± 3.1	10.3 ± 3.0
ABC Scale	75.3 ± 16.8	75.0 ± 17.6	75.5 ± 16.9

*ABC Scale* Activities-specific Balance Confidence Scale, *BMI* body mass index, *CI* confidence interval, *FES-I* Falls Efficacy Scale International, *ITT* intention-to-treat, *LLFDI* Late Life Function and Disability Instrument, *max* maximal, *MoCA* Montreal Cognitive Assessment, *No*. Number, *p.p*. per person, *SD* standard deviation, *TUG* Timed Up-and-Go

### Primary outcomes

#### Combined endpoint: Falls adjusted for physical activity

**Fig. 2:**
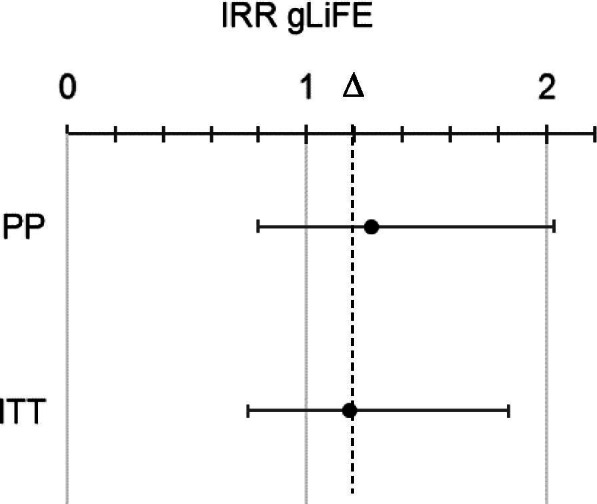
Observed treatment differences in incidence risk ratio (IRR) at 6 months between LiFE (reference) and gLiFE with non-inferiority ∆ set at 20%; PP: per protocol; ITT: intention-to-treat

Compared to LiFE, gLiFE had an incidence rate ratio of 1.07 (95% CI: 0.73; 1.57) at baseline and of 1.27 (95% CI: 0.80; 2.03) at 6 months according to PP analysis. When applying ITT analysis, IRRs at baseline (1.04; 95% CI: 0.72; 1.50) and 6 months (1.18; 95% CI: 0.75; 1.84) were smaller (Fig. 2). In both cases, non-inferiority was inconclusive due to upper confidence intervals crossing the 20% margin (∆) at 6 months. This means there was a non-significant difference in the risk of experiencing a fall for gLiFE compared to LiFE participants. When subtracting the initial baseline difference of 7.3% (4.1%), the changed IRR between both groups remains at 20% (14%).

#### Intervention costs

Under study conditions the total intervention costs per participant amounted to €350.10 for LiFE compared to €229.93 for gLiFE. This corresponded to a cost advantage of €121.17 for gLiFE. This cost advantage mainly resulted from a difference in personnel costs for trainers. In contrast, costs for room rent and travel expenses were marginally higher for gLiFE than LiFE with €23.45 and €17.92, respectively. Under “real world” assumptions, average costs per gLiFE participant were €120.58 compared to €332.08 per LiFE participant, resulting in a cost difference of €211.51 in favour of gLiFE. Again, this was mainly due to a difference in personnel costs. Intervention costs are summarized in table S2.

### Secondary outcomes

#### Physical activity

Both groups increased their amount of steps/day between baseline and follow-up (Table 2). The increase in the gLiFE group was significantly larger than in the LiFE group in both PP (gLiFE to LiFE: + 880 steps; CI 252; 1,509) and ITT (gLiFE to LiFE: + 844; CI 176; 1,512) analyses. gLiFE participants increased their steps/day from 5,530 (SE 237); in LiFE, the increase from 5,880 (SE 255) was about a third (35,5%) of the increase in the gLiFE group according to ITT (PP: 30,5%).

**Table 2:** Baseline and 6-month secondary outcome data and between-group comparison

mean ± SE	LiFE		gLiFE		Between-group difference (95% CI) gLiFE vs. LiFE	
	PP	ITT	PP	ITT	PP	ITT
Mean steps/day						
Baseline	5,880 ± 255	5,778 ± 250	5,530 ± 237	5,538 ± 234		
Post	6,266 ± 254	6,242 ± 257	6,797 ± 264	6,847 ± 257		
*Change*	*386* ± *227*	*465* ± *257*	*1,266* ± *213*	*1,309* ± *225*	*880 (252; 1,509)* *p* = *.007*	*844 (176; 1,512)* *p* = *.015*
LLFDI function						
Baseline	57.4 ± 0.68	57.4 ± 0.64	57.4 ± 0.63	57.3 ± 0.64		
Post	58.9 ± 0.75	58.9 ± 0.76	59.1 ± 0.76	59.2 ± 0.77		
*Change*	*1.5* ± *0.48*	*1.5* ± *0.55*	*1.7* ± *0.51*	*1.9* ± *0.57*	*0.18 (-1.23; 1.58)* *p* = *.807*	*0.31 (-1.33; 1.95)* *p* = *.710*
LLFDI frequency						
Baseline	49.5 ± 0.34	49.3 ± 0.33	49.8 ± 0.38	49.5 ± 0.35		
Post	50.3 ± 0.41	50.1 ± 0.40	50.2 ± 0.41	50.0 ± 0.42		
*Change*	*0.8* ± *0.29*	*0.8* ± *0.30*	*0.4* ± *0.30*	*0.48* ± *0.34*	*-0.47 (-1.32; 0.38)* *p* = *.279*	*-0.29 (-1.25; 0.66)* *p* = *.546*
LLFDI disability						
Baseline	71.8 ± 1.03	71.7 ± 0.99	69.3 ± 0.94	69.6 ± 0.93		
Post	70.8 ± 1.01	70.8 ± 1.05	71.3 ± 1.14	71.2 ± 1.10		
*Change*	*-1.0* ± *1.05*	*-0.9* ± *1.10*	*1.9* ± *1.04*	*1.5* ± *1.08*	*2.87 (-0.05; 5.79)* *p* = *.054*	*2.40 (-0.71; 5.50)* *p* = *.132*
FESI						
Baseline	10.4 ± 0.27	10.4 ± 0.25	10.3 ± 0.25	10.3 ± 0.24		
Post	9.7 ± 0.23	9.6 ± 0.23	9.4 ± 0.22	9.5 ± 0.22		
*Change*	*-0.8* ± *0.24*	*-0.8* ± *0.24*	*-0.9* ± *0.21*	*-0.8* ± *0.22*	*-0.14 (-0.76; 0.49)* *p* = *.669*	*0.00 (-0.62; 0.62)* *p* = *.996*
ABC						
Baseline	74.7 ± 1.48	75.0 ± 1.41	75.6 ± 1.36	75.5 ± 1.36		
Post	78.8 ± 1.25	79.1 ± 1.21	77.7 ± 1.31	77.0 ± 1.30		
*Change*	*4.1* ± *1.16*	*4.1* ± *1.14*	*2.1* ± *1.27*	*1.5* ± *1.32*	*-1.94 (-5.31; 1.43)* *p* = *.259*	*-2.62 (-6.09; 0.84)* *p* = *.138*
30 s chair rise						
Baseline	9.1 ± 0.32	9.2 ± 0.31	9.0 ± 0.27	9.0 ± 0.27		
Post	9.6 ± 0.36	9.5 ± 0.34	10.0 ± 0.38	10.0 ± 0.40		
*Change*	*0.49* ± *0.31*	*0.38* ± *0.30*	*1.10* ± *0.31*	*1.00* ± *0.34*	*0.56 (-0.26; 1.38)* *p* = *.180*	*0.63 (-0.25; 1.51)* *p* = *.164*
8 level balance scale						
Baseline	4.2 ± 0.13	4.2 ± 0.12	4.4 ± 0.12	4.4 ± 0.11		
Post	4.2 ± 0.13	4.2 ± 0.13	4.6 ± 0.14	4.6 ± 0.14		
*Change*	*0.0* ± *0.12*	*0.0* ± *0.13*	*0.2* ± *0.14*	*0.25* ± *0.13*	*0.21 (-0.58; 0.15)* *p* = *.248*	*0.20 (-0.17; 0.58)* *p* = *.290*
Gait speed normal						
Baseline	1.04 ± 0.02	1.03 ± 0.02	1.05 ± 0.02	1.03 ± 0.02		
Post	1.07 ± 0.02	1.07 ± 0.02	1.07 ± 0.02	1.06 ± 0.02		
*Change*	*0.04* ± *0.01*	*0.04* ± *0.01*	*0.02* ± *0.02*	*0.03* ± *0.02*	*-0.01 (-0.05; 0.02)* *p* = *.458*	*-0.01 (-0.05; 0.03)* *p* = *.651*
Gait speed fast						
Baseline	1.38 ± 0.02	1.37 ± 0.02	1.44 ± 0.03	1.43 ± 0.03		
Post	1.40 ± 0.03	1.39 ± 0.03	1.43 ± 0.03	1.41 ± 0.03		
*Change*	*0.02* ± *0.02*	*0.02* ± *0.02*	*-0.01* ± *0.02*	*-0.02* ± *0.02*	*-0.03 (-0.08; 0.02)* *p* = *.243*	*-0.04 (-0.09; 0.02)* *p* = *.212*

#### Fall outcomes

According to ITT analyses, 109 falls (PP: 100) were recorded; 49 in LiFE and 60 in gLiFE participants between baseline and 6-month follow-up. Of these, 29 persons fell once in LiFE and 27 in gLiFE; 9 persons in LiFE and 15 in gLiFE fell at least twice (maximum of 4 falls in each group), that is, there were 38 fallers in LiFE and 42 in gLiFE. The incidence of falls per half person year (ITT) in LiFE was 0.30 (SE 0.05) and 0.40 (SE 0.06) in gLiFE (PP; gLiFE: 0.41, SE 0.06; LiFE: 0.30, SE 0.05). The time to event (first fall) and observation time were similar in both groups (median = 180 days). Comparing the number of falls per half person year at baseline and 6 months, the incidence decreased about 37% (0.65 to 0.41) in gLiFE and 55% in LiFE (0.66 to 0.30) (ITT). Falls entailed mild (75%), moderate (18%), and severe (7%) consequences as per definition of Schwenk et al. [26].

#### Motor capacity

Results of motor capacity assessments are shown in Table 2. No significant differences in changes of motor capacity between groups were found for any of the measures, although improvement in gLiFE was larger in 30 s. chair rise (+ 1.00 vs. + 0.38 in LiFE) and 8 level balance scale (+ 0.25 vs. ± 0.00 in LiFE). LiFE participants showed somewhat larger improvement in comfortable gait speed (gLiFE + 0.03 m/s vs. LiFE + 0.04 m/s) and fast gait speed (gLiFE -0.02 vs. LiFE + 0.02). PP analyses did not alter these findings.

#### Functional (dis-)ability

According to LLFDI results, increase in both groups in the functional domain was comparable. Self-perceived function increased slightly from 57.3 (SE 0.6) to 59.2 (SE 0.8) in gLiFE and from 57.4 (SE 0.6) to 58.9 (SE 0.8) (ITT; mean difference 0.3; 95% CI: -1.33; 1.95; *p* = 0.710). Self-perceived disability improved from 69.6 (SE 0.9) to 71.2 (SE 1.1) in gLiFE and decreased from 71.7 (SE 1.0) to 70.8 (SE 1.0) in LiFE (ITT; mean difference 2.4; 95% CI: -0.71; 5.50) in favor of gLiFE. PP analysis did not alter these findings.

#### Adherence

Both groups had a high proportion of completers; 99% of LiFE and 88% of gLiFE participants took part in more than 75% of the sessions. Under ITT stipulations, those numbers expectedly dropped to 91 and 78%, respectively. With 7.8 sessions on average, gLiFE participants attended significantly less sessions than LiFE participants with 8.7 sessions (PP: 95% CI: -0.68; -1.12). ITT analysis did not change this finding. Duration adherence according to EARS scores were significantly lower in the gLiFE group: 14.9 in gLiFE compared to 16.0 in LiFE (95% CI: -0.01; -2.18). Again, ITT analysis did not affect this finding.

#### Fear of falling and balance confidence

Fear of falling decreased in both groups to a comparable level. In gLiFE, it dropped from 10.3 to 9.5 (change of -0.8; SE 0.2); in LiFE, it dropped from 10.4 to 9.6 (change of -0.8; SE 0.2). The between-group difference in this change was not significant (95% CI: -0.62; 0.62). Balance confidence values (ABC scale) were increased in both groups, however, differences in these changes were not significant (-2.62; 95% CI: -6.09; 0.84). PP analyses did not alter these findings.

## Discussion

In the LiFE-is-LiFE trial it was investigated (1) whether gLiFE as a group format of the LiFE program was non-inferior to the individually delivered, original LiFE format in terms of activity-adjusted fall risk, and (2) whether both formats differed in their effect on PA and several function- and adherence-related outcomes. To the best of our knowledge, there has not been any direct comparison of a group format and an individually delivered format of the same intervention program so far; even less one including an economic evaluation.

### Primary outcomes

For the main outcome, activity-adjusted fall risk operationalized as IRR, non-inferiority was not confirmed as the upper bound of the two-sided 95% confidence interval crossed the predefined 20% non-inferiority margin. Per definition, this means that non-inferiority of gLiFE is ‘inconclusive’ [18]. One reason for this could be that we used 6-month instead of 12-month data, and that differences at 6 months are of different nature than at 12 months. As there are less fall events within 6 than within 12 months, there is a higher risk of random error which could have influenced the point estimate. From an intervention perspective, the LiFE group was under more direct and closer individual supervision than gLiFE participants during the intervention phase, which might come with more problems to independently conduct and adapt LiFE activities as compared to gLiFE participants in the long term. Unlike LiFE participants who had direct suggestions and support, gLiFE participants had learnt to implement LiFE activities in their own way at home right from the start. We also see that the mean IRR is very close to the 20% margin, suggesting that the actual difference could be close to these 20%.

Comparing the reduction in overall incidence of falls between baseline and follow-up, both groups in the present trial reduced their fall incidence to a great extent. Despite the fact that fall incidence was already low in our sample at baseline, these reductions were greater (37% gLiFE; 55% LiFE) than in the LiFE group in the reference trial by Clemson et al. (22%) [14].

Regarding the second primary outcome, intervention costs of several exercise-based fall prevention programs have already been determined as part of economic analyses [35]. However, such economic analyses have not been performed for the LiFE program, yet, despite the high effectivity of this program for reducing falls and increasing PA [14]. This is of interest for potential payers of the intervention in case of implementation. Our findings highlight that gLiFE was associated with lower intervention costs compared to LiFE while at the same time reducing falls in both formats, making it an attractive alternative from a payer’s and individual's perspective. The size of the cost advantage depended primarily on the ratio of participants to trainers. Therefore, the cost advantage was particularly pronounced in the "real world" scenario, which assumed a higher number of participants per group. Moreover, the total intervention costs per participant depended on the trainers’ salary or the number of groups each trainer/trainer pair supervises. Hence, there is not only one possible scenario of the "real world", but the assumptions made in this study were found to be the most realistic by intervention experts. For an informed recommendation regarding implementation, other health-care utilization costs beside intervention costs must be examined in relation to the health effects. An economic evaluation regarding the cost-effectiveness of gLiFE will assist in making implementation recommendations and is part of further analyses [36].

### Secondary outcomes

In general, the main idea of the LiFE program–to promote safe PA and simultaneously improve motor function [15]–was confirmed. PA, operationalized as walking activity for our study purpose, was increased in both groups. With 23%, this increase was significantly higher in gLiFE than in LiFE with 7%. Walking has been identified as key factor in promoting PA and health [37] and steps/day are a tangible activity goal for both participants and trainers [38]. Increased walking activity over more than 1,000 steps/day comes with lower risk of all-cause mortality as well as cardiovascular disease morbidity and mortality [39]. Hence, on average gLiFE seems better suited to evoke activity-related health benefits. This is further supported in that other studies have shown much lower pre-to-post intervention changes in steps/day, averaging around 800 steps change in older, mainly community-dwelling adults [40]. With an average between 5,500 and 5,800 steps/day in both groups at baseline, our sample was slightly more active than in studies with large samples of men and women of similar age [7, 41], indicating at least moderate activity levels. The examination of mechanisms of action in LiFE and gLiFE do not provide evidence for the superiority of gLiFE in affecting psychological determinants which are assumed to translate into behavioral changes [42]. However, it is possible that gLiFE participants profited of the group program in a way we did not capture with our measurements, e.g., through comparison with peers.

For other secondary outcomes, there were no significant differences in changes over six months between both groups. Descriptive data showed somewhat larger improvement over 6 months in gLiFE for LLFDI disability, but not for any other secondary measure. The difference between comfortable and fast gait speed at baseline indicates that there is a notable reserve in functional capacity in our sample. Taking into account that gLiFE participants had significantly lower attendance rates and duration adherence, it seems that the ‘dose’ given to gLiFE participants was still sufficient to achieve effects comparable to LiFE.

The LiFE intervention was delivered similarly to the reference trial by Clemson and colleagues [14], but underwent small organizational changes, which were needed to align LiFE and gLiFE contents in our study (this is further discussed elsewhere [17, 19]). Duration adherence at 6 months according to EARS was medium to good in both groups with mean values confirming adherence to their plans. LiFE participants had a higher adherence at 6 months, which could be due to higher intervention attendance rates. The fact that attendance was significantly higher in LiFE than in gLiFE was not surprising as the group session schedule was predetermined and not movable whereas individual appointments in LiFE could easily be moved according to participants’ requirements. One factor that might have boosted effectiveness of gLiFE is social support by other group members, which was found to be supportive of engagement in exercise and PA [43]. According to an extensive review on exercise interventions to prevent falls, however, there is no difference in effect based on intervention format (group vs. individual) [44]. Further analyses are needed to determine which characteristics of both formats are responsible for differences in both groups’ outcomes.

### Strengths and limitations

Results of the present study need to be interpreted in the light of several strengths and limitations. Non-inferiority trials are becoming more frequent, aiming to establish interventions’ non-inferiority over another treatment [19]. Instead of developing new interventions which then have to undergo extensive scientific evaluation, it seems worth looking at already established intervention programs such as LiFE. By adapting or refining existing interventions, their feasibility and cost-effectiveness could be improved, which in turn would come with advantages for participants and payers equally. The LiFE program has the benefit of being carried out at participants’ homes, which entails fewer burdens to physical exercise than conventionally delivered structured exercise. Many of those burdens, especially those being highlighted by older adults [45] do not apply in the LiFE program (e.g., bad weather, lack of time). Having shown that both modes of delivery come with meaningful health benefits such as enhanced PA, gLiFE could now be made available also to those who prefer company of others. At the same time, those who prefer being on their own can be served as well. Another strength is that activity-adjusted falls were assessed using highly reliable methods. For fall documentation, participants completed fall calendars [46]; PA was assessed using highly reliable sensors over a full week with very few incomplete measurements (< 2%). Moreover, we followed the extended consort statement of 2010 for non-inferiority trials, thus abiding by clear reporting and interpretation standards. Lastly, data analyses were carried out for both PP and ITT including multiple imputation [32].

Despite many strengths, some limitations are to be considered. As pandemic circumstances had a strong impact on older adults’ habitual PA and overall movement behavior [47] it is expected that 12-month follow-up data were highly biased. COVID-19 pandemic regulations began shortly after completion of the 6-month assessments, and about one third of the participants were not assessed regularly as part of 12-month follow-up within the specified time window. Attempts were therefore made to follow up any unscheduled assessment after re-opening of public structures following the lockdown. Therefore, we chose to deviate from the study protocol by evaluating non-inferiority based on 6-month instead of 12-month data. Moreover, pre-baseline falls data were assessed retrospectively for 6 months. Comparing 6-month follow-up fall data with baseline falls therefore is to be done very cautiously due to the different standard and sources of bias in falls assessment [24]. Due to the established effectiveness of the LiFE program [14], no control group was included in this trial. Natural progression of IRRs without intervention therefore cannot be quantified. Compared to the Australian LiFE study [14], the present sample was somewhat younger (-4 years), had a higher proportion of women (+ 14%) and less falls in the past (0.66 per person and half year compared to 2.13 per person and year in Clemson et al.), which limits comparability with our findings.

## Conclusions

Non-inferiority of gLiFE’s reduction of falls compared to LiFE was inconclusive, while its increase in walking activity was significantly higher than in LiFE, which shows its large potential especially in promoting PA. In the light of lower intervention costs compared to LiFE, gLiFE is an alternative from a payer’s and individual's perspective. Our results suggest that both formats come with important effects and advantages, and that individuals should be given the opportunity to choose between both formats depending on their individual goals.

## Supplementary Information

**Additional file 1.** Data and assumptions for the calculation of Intervention costs by scenario

**Additional file 2.** Intervention costs per participant for gLiFE and LiFE by scenario and cost category

**Additional file 3. **CONSORT Statement 2006 - Checklist for Non-inferiority and Equivalence Trials

## Data Availability

The datasets used and/or analyzed during the current study are available from the corresponding author on reasonable request.

## Abbreviations

ABC Scale: Activities-specific balance confidence scale
BMI: Body mass index
CI: Confidence interval
EARS: Exercise Adherence Rating Scale
FES-I: Falls Efficacy Scale International
gLiFE: Group-based Lifestyle-integrated Functional Exercise
IRR: Incidence rate ratio
ITT: Intention-to-treat
LiFE: Lifestyle-integrated Functional Exercise
LLFDI: Late-life function and disability instrument
MoCA: Montreal cognitive assessment
No.: Number
PA: Physical activity
p.p.: Per person
PP: Per protocol
SD: Standard deviation
SE: Standard error
TIDieR: Template for intervention description and replication
TUG: Timed Up-and-Go test
