# Supplementary material for: Lifestyle-integrated functional exercise to prevent falls and promote physical activity: Results from the LiFE-is-LiFE randomized non-inferiority trial
Source: Int J Behav Nutr Phys Act. 2021 Sep 3;18:115. doi: 10.1186/s12966-021-01190-z (PMC8414469; doi:10.1186/s12966-021-01190-z)
Supplement: Supplementary file 1 — Additional file 1. Data and assumptions for the calculation of Intervention costs by scenario [file 12966_2021_1190_MOESM1_ESM.docx]

Additional file 1: Data and assumptions for the calculation of Intervention costs by scenario

|  | **study conditions** | **„real world“** |
| --- | --- | --- |
| **Trainer workshop** |  |  |
| duration | 3 days | 3 days |
| salary group coaches conducting the workshop | TVöD^1^ E13 | TVöD E13 |
| number and salary level of the trainers | 8 trainers  (TVöD E10 or E13) | 20 trainers  (TVöD E8) |
| **LiFE sessions** |  | |
| average duration (including preparation and travelling time) | 111 min | 120 min |
| (average) number of sessions per participant | 6.49 | 7 |
| (average) number and duration of “booster phone calls” | 1.66; 27 min | 2.00; 30 min |
| number of material sets per trainer | 1 | 1 |
| **gLiFE sessions** |  | |
| average duration (including preparation and travelling time)^2^ | 180 min | 150 min |
| (average) initial number of participants per group | 10.2 (average number of participants per session due to study drop out and occasional non-participation: 7.9) | 12.0  (assumption: sessions have to be paid regardless of participation) |
| (average) number and duration of “booster phone calls” | 1.65, 29 min | 2.00, 30 min |
| room rent per session | 50€ for 8 out of 15 groups  (study centre Stuttgart) | 0€ |
| number of material sets per trainer pair | 1 | 1 |
| **Other assumptions** |  |  |
| number of sessions per trainer (pair) per week | not relevant – 8 trainers taught the program to a similar number of LiFE/gLiFE participants (n=156 & n=153) over the study period | LiFE: 15 (30h); gLiFE: 12 (30h) |
|  |  |  |
| ***Study conditions****: calculation of intervention costs as incurred during the study*  *“****Real world****”: calculation of intervention costs based on modified assumptions reflecting more realistic conditions in case the program is implemented than study conditions*  ***^1^TVöD****: civil service collective agreement*  ***^2^****In one study centre, trainers had travelling time to an external room where the group sessions were held; in the “real world” scenario, no travelling time is taken into account for gLiFE (assumption: a suitable room is available at the workplace)* | | |
