# Supplementary material for: Lifestyle-integrated functional exercise to prevent falls and promote physical activity: Results from the LiFE-is-LiFE randomized non-inferiority trial
Source: Int J Behav Nutr Phys Act. 2021 Sep 3;18:115. doi: 10.1186/s12966-021-01190-z (PMC8414469; doi:10.1186/s12966-021-01190-z)
Supplement: Supplementary file 2 — Additional file 2. Intervention costs per participant for gLiFE and LiFE by scenario and cost category [file 12966_2021_1190_MOESM2_ESM.docx]

Additional File 2: Intervention costs per participant for gLiFE and LiFE by scenario and cost category

| **Study conditions** | | | **„Real world“** | | |
| --- | --- | --- | --- | --- | --- |
| ***gLiFE*** | ***LiFE*** | ***Difference*** | ***gLiFE*** | ***LiFE*** | ***Difference*** |
| Trainer workshop (including personnel and material costs) | | | | | |
| € 17.69 | € 17.69 | € 0.00 | € 6.55 | € 31.44 | € -24.89 |
| Personnel costs training session and ‘booster phone calls’ | | | | | |
| € 138.58 | € 301.96 | € - 136.38 | € 66.75 | € 269.65 | € -202.90 |
| Room rent training session | | | | | |
| € 23.45 | € 0.00 | € + 23.45 | € 0.00 | € 0.00 | € 0.00 |
| Material costs training session | | | | | |
| € 31.29 | € 30.45 | € + 0.84 | € 29.35 | € 30.99 | € -1.64 |
| Average travel expenses (participants) | | | | | |
| € 17.92 | € 0.00 | € + 17.92 | € 17.92 | € 0.00 | € + 17.92 |
| **Total** | | | | | |
| **€ 228.93** | **€ 350.10** | **€ - 121.16** | **€ 120.58** | **€ 332.08** | **€ - 211.51** |
| ***Study conditions****: calculation of intervention costs as incurred during the study*  *“****Real world****”: calculation of intervention costs based on modified assumptions reflecting more realistic conditions in case the program is implemented than study conditions* | | | | | |
